# Discharge-time prediction of 1-month posttraumatic stress symptom severity (PCL-5) after mechanical ventilation using a dual-attention 1D-CNN: Development and validation

**DOI:** 10.1371/journal.pmen.0000629

**Published:** 2026-06-09

**Authors:** Qingqing Xia, Wei Zhang, Jinqiang Zhuang, Ming Zhu, Lei Wu

**Affiliations:** 1 Jiangsu University Jingjiang College, Jiangsu University, Zhenjiang, China; 2 The Affiliated Hospital of Yangzhou University, Yangzhou University, Yangzhou, China; Instituto Federal do Maranhão: Instituto Federal de Educacao Ciencia e Tecnologia do Maranhão, BRAZIL

## Abstract

Mechanical ventilation (MV) in the emergency intensive care unit (EICU) may expose patients to traumatic experiences that increase the risk of early post-discharge posttraumatic stress symptoms. A discharge-time tool for estimating symptom severity could support early risk stratification and follow-up planning. This single-center prospective observational cohort study enrolled mechanically ventilated EICU patients between July 17, 2025 and January 10, 2026, with 1-month follow-up. Pre-discharge demographic, clinical, and psychosocial variables were collected, and the Posttraumatic Stress Disorder Checklist for DSM-5 (PCL-5) assessments were completed at 1 month. After data cleaning, 500 participants were retained and randomly divided into a development set (n = 400) and an independent test set (n = 100). Based on univariate screening, clinical relevance, and data completeness, 11 predictors were entered into a dual-attention 1D-CNN multi-output regression model to predict the PCL-5 total score and four symptom-cluster scores, incorporating a constraint term enforcing the relationship between the total score and the sum of the cluster scores. Mean PCL-5 total was 38.0 ± 9.1; 70.8% of participants met the screening threshold for clinically significant posttraumatic stress symptoms (PCL-5 ≥ 33). The CNN-Attention model achieved R^2^ = 0.804 for total score on the test set (RMSE = 2.164; MAE = 1.935; MAPE = 7.43%) and R^2^ = 0.817–0.929 for clusters, outperforming comparator models. A discharge-time CNN-Attention model based on routinely available variables showed good performance in predicting early post-discharge PTSD symptom burden and symptom profiles after MV. Further multicenter studies are needed to externally validate the model and assess its clinical utility.

## 1 Introduction

### 1.1 Research background

The EICU plays a crucial clinical role in the early identification and rapid management of emergency and critically ill conditions. Patients admitted to the EICU often have rapidly progressing conditions and frequently require invasive life-support interventions. Within a short period, they may undergo multiple high-stress experiences, including tracheal intubation, sedation and analgesia, physical restraint, delirium, and separation from family members, which can contribute to a substantial psychological trauma burden [1].

In China, approximately 50%–70% of critically ill patients require mechanical ventilation [2]. Although mechanical ventilation is life-saving, it may also be associated with distressing physical and psychological experiences. In particular, MV is often accompanied by pronounced feelings of loss of control, suffocation, distressing traumatic memories, and perceived threats to life, which may increase the risk of subsequent posttraumatic stress symptoms [3].

Psychological problems among ICU survivors constitute a major component of Post-Intensive Care Syndrome (PICS) [4], and posttraumatic stress symptoms are particularly important because of their association with functional impairment and prolonged recovery. Core symptom clusters include intrusion, avoidance, negative alterations in cognition and mood, and alterations in arousal and reactivity [5]. These symptoms may persist for months or even years and adversely affect long-term recovery [6].

Among mechanically ventilated patients, posttraumatic stress symptoms are not limited to the months following discharge. A substantial proportion of patients exhibit significant symptom burden during hospitalization or in the early post-discharge period (e.g., within 1 month). Without timely identification and intervention, these symptoms may become persistent and more difficult to address [7]. In current EICU practice, care remains primarily focused on life support and organ function recovery, while psychological risk assessment is often deferred until after discharge. Because specialized mental health resources are limited, a discharge-time tool based on routinely available information may help estimate early post-discharge PTSD symptom severity and support risk stratification, follow-up planning, and efficient allocation of psychological care.

### 1.2 Current status of domestic and international research

#### 1.2.1 Populations and Incidence.

With the progressive improvement in survival rates among critically ill patients, psychological health outcomes have gained growing attention from researchers worldwide. Numerous studies have reported the prevalence of psychological disorders, including anxiety, depression, and PTSD, among ICU survivors. However, the majority of these studies have been conducted in Europe and North America, and data from Asian countries, including China, remain limited [8].

Available evidence suggests that the prevalence of PTSD or clinically significant posttraumatic stress symptoms among mechanically ventilated ICU patients ranges from 17.7% to 45.5% [9]. In contrast, research specifically focusing on PTSD in mechanically ventilated EICU patients remains scarce. A multicenter study conducted by Unoki T et al. [7], involving patients discharged home from 12 ICUs in Japan, demonstrated that critically ill patients admitted on an unplanned basis exhibited significantly higher PTSD symptom burden and greater symptom severity compared with those admitted electively. Similarly, a follow-up study by Shima N [10] of adult ICU patients admitted via the emergency department reported that the prevalence of PTSD or clinically significant posttraumatic stress symptoms at 3 months after discharge reached up to 20%. Considerable heterogeneity exists among these studies, which may be attributable to variations in baseline population characteristics, differences in follow-up timing, and the use of diverse assessment instruments.

#### 1.2.2 Assessment and diagnosis.

Among available methods, clinician-administered interviews and self-report scales have become the preferred tools for PTSD assessment in clinical and research settings.

Internationally, the evolution of PTSD assessment instruments has closely paralleled updates to DSM diagnostic criteria, resulting in a comprehensive and well-established system of scales. The Posttraumatic Stress Disorder Checklist for DSM-5 (PCL-5) is a widely used 20-item self-report instrument aligned with the four DSM-5 symptom clusters and is commonly used to assess PTSD symptom severity in research and clinical follow-up [11]. The Clinician-Administered PTSD Scale (CAPS) is widely recognized as the reference standard for PTSD diagnosis [12].

#### 1.2.3 Influencing factors and prediction models.

Extensive investigations have been conducted worldwide to identify factors associated with posttraumatic stress symptoms among critically ill patients, providing an essential foundation for the development of predictive models. These associated factors can be broadly categorized into demographic factors, disease- and treatment-related factors, and psychological factors.

(1) Demographic factors

Demographic characteristics such as sex, age, educational attainment, marital status, employment status, and social support have been reported to be associated with posttraumatic stress symptoms after critical illness [13–18]. However, findings across studies remain heterogeneous, and the strength and direction of these associations are not fully consistent.

(2) Disease- and treatment-related factors

Disease characteristics and treatment-related exposures may also be associated with posttraumatic stress symptoms in critically ill patients, partly by shaping the intensity and nature of the traumatic medical experience. Evidence suggests that prior medical history may affect posttraumatic stress outcomes in critically ill populations. Patients with chronic comorbidities may be more vulnerable to adverse posttraumatic stress outcomes when confronted with additional stressful events [19]. Importantly, characteristics of the critical illness experience itself may contribute to subsequent posttraumatic stress symptoms.

The association between disease severity indicators, such as APACHE II, and posttraumatic stress outcomes remains inconsistent across studies [15,20]. One possible explanation is that physiological severity and subjective traumatic experience are related but not equivalent constructs.

Treatment-related exposures during mechanical ventilation, including sedation and analgesia, have also been associated with posttraumatic stress outcomes, although findings vary across studies and underlying mechanisms remain unclear [21,22].

Longer duration of mechanical ventilation has been associated with higher levels of posttraumatic stress symptoms after discharge [23,24]. Longer ventilation duration may also increase the risk of delirium, which has been linked to fragmented, delusional, and distressing ICU memories and may thereby contribute to subsequent posttraumatic stress symptoms [25,26]. However, the direction and mechanisms of these associations remain debated.

(3) Psychological factors

Psychological factors, particularly pre-existing mental health problems and acute stress responses, are also important correlates of posttraumatic stress outcomes in critically ill patients. Substantial evidence indicates that pre-existing mental disorders are associated with a higher risk of posttraumatic stress symptoms [27]. One possible explanation is that prior mental illness may reduce stress tolerance and coping capacity, thereby increasing vulnerability to posttraumatic stress symptoms during critical illness.

Compared with chronic psychological vulnerability, acute psychological stress states demonstrate stronger predictive value for subsequent posttraumatic stress symptoms. Wade DM et al. [20] reported that acute psychological factors were among the strongest and potentially modifiable predictors of posttraumatic stress outcomes, beyond disease severity and chronic health status. In mechanically ventilated patients, abrupt loss of control and dependence on life-support devices may intensify acute stress and contribute to subsequent posttraumatic stress symptoms.

Posttraumatic stress symptoms often co-occur with anxiety and depression in ICU survivors [8]. This overlap highlights the importance of incorporating psychological variables into prediction-oriented analyses rather than considering posttraumatic stress symptoms in isolation. Accordingly, the present study included anxiety and depression in the candidate predictor set.

(4) Prediction models

Early identification of patients at elevated risk may support follow-up planning and targeted assessment, motivating the development of prediction models for posttraumatic stress outcomes. Previous research on posttraumatic stress outcomes in critically ill populations has predominantly focused on associated factors, often relying on traditional regression approaches and binary endpoints [28]. Such approaches present two notable limitations. First, psychological outcomes in critically ill patients are influenced by multiple interacting dimensions, including demographic characteristics, disease severity, treatment-related factors, complications, and subjective experiences. The interactions among these variables are complex and frequently non-linear, making them difficult to capture using conventional linear modeling assumptions. Second, binary outcome prediction alone is insufficient to support fine-grained clinical management of symptom severity and limits the ability to develop differentiated follow-up and intervention strategies.

Deep learning models offer the ability to capture complex, non-linear relationships between multidimensional clinical data and psychological outcomes without reliance on strong linear assumptions. In particular, convolutional neural networks (CNNs) can automatically extract latent patterns from high-dimensional features, while attention mechanisms further enhance model performance by selectively emphasizing key features and improving sensitivity to clinically relevant information. However, CNN-Attention–based models for predicting continuous PCL-5 total and symptom-cluster scores in mechanically ventilated EICU patients remain limited. Accordingly, this study developed and internally validated a CNN-Attention model using routinely available discharge-time variables to estimate PTSD symptom severity at 1 month after discharge. This approach was intended to support early risk stratification and follow-up planning.

### 1.3 Research objectives and significance

This study aimed to develop and internally validate a CNN-Attention multi-output regression model for estimating the PCL-5 total score and four symptom-cluster scores at 1 month after discharge among young and middle-aged mechanically ventilated EICU patients. By using routinely available demographic, clinical, treatment-related, and psychological variables, the model was intended to provide a quantitative tool for early risk stratification and follow-up planning.

This study has potential methodological and clinical relevance. From a theoretical perspective, a CNN-Attention deep learning framework was applied to the multidimensional prediction of PTSD symptom severity in young and middle-aged mechanically ventilated EICU patients, addressing the limited application of CNN-Attention models to PTSD symptom severity prediction in this clinical population. Furthermore, the incorporation of a loss function design with mathematical constraints among multiple output variables may provide a methodological reference for optimizing multi-output regression models.

From a clinical practice perspective, psychological assessment is not routinely prioritized during the early management of emergency and critically ill patients in China. At the same time, limited mental health resources make comprehensive psychological assessment difficult to implement for all critically ill patients. In addition, PTSD diagnosis cannot rely on a single assessment scale and generally requires multidimensional evaluation by trained professionals, which further increases clinical workload. By using the total PCL-5 score as the primary predictive outcome, the proposed model may help clinicians obtain an early estimate of symptom burden and identify patients who may benefit from further psychological assessment or referral. This approach may support early risk stratification and follow-up planning, while its clinical utility should be further evaluated in future studies.

## 2 Data collection and preprocessing

### 2.1 Ethical statement

This study was reviewed and approved by the Ethics Committee of the Affiliated Hospital of Yangzhou University (approval number: 2025-YKL07-K02). Prior to participation, all patients were informed of the purpose, significance, and procedures of the study. Participation was entirely voluntary, and written informed consent was obtained from each participant. All participants were informed of their right to withdraw from the study at any time without any consequences.

### 2.2 Study population

This study employed a single-center prospective cohort design. Young and middle-aged patients receiving mechanical ventilation in the EICU of the Affiliated Hospital of Yangzhou University were consecutively enrolled between July 17, 2025, and January 10, 2026, and completed a follow-up survey 1 month after discharge.

The inclusion criteria were as follows: ① age between 18 and 59 years; ② duration of mechanical ventilation exceeding 24 hours; ③ clear mental status after weaning and ability to cooperate with the required assessments; ④ absence of severe cognitive impairment; and ⑤ no acute psychiatric episodes or severe psychiatric symptoms that would preclude assessment (patients with a history of mental illness were eligible if their condition was stable and they were able to complete the assessment; such history was recorded as a predictive feature).

Exclusion criteria included the presence of severe organic brain lesions affecting cognitive or psychological evaluation, as well as treatment withdrawal or transfer to another institution that prevented completion of the full study protocol.

### 2.3 Data collection and sources of variables

Previous studies have demonstrated that questionnaire response rates decline as follow-up duration increases, and that prognosis among critically ill patients is inherently uncertain, with prolonged follow-up increasing the risk of loss to follow-up [10,29]. Moreover, the PCL-5 scale assesses symptoms experienced over the preceding month, and prompting patients to recall intensive care experiences too early may exacerbate psychological distress. Accordingly, a staged data collection strategy was implemented. A general information questionnaire was completed prior to discharge, while the PCL-5 scale was administered at 1 month after discharge via WeChat or email, in order to ensure data completeness and patient safety.

The general information questionnaire was structured into three components. The first component comprised demographic data, with basic patient information extracted from the EICU case management system, including age, sex, and prior medical history. The second component included clinical diagnosis- and treatment-related variables, such as duration of mechanical ventilation, types and dosages of sedative medications, and cumulative days of delirium. The third component assessed patients’ subjective experiences and psychological status, covering aspects such as disease-related family burden, levels of anxiety and depression, and the integrity of memories during mechanical ventilation. These data were obtained through face-to-face interviews with patients and their family members after weaning and extubation, once patients had regained a clear mental status.

Anxiety and depression symptom severity prior to discharge was evaluated using the Hospital Anxiety and Depression Scale (HADS). The HADS was developed by Zigmond and Snaith in 1983 [30] and consists of two subscales: anxiety (HADS-A) and depression (HADS-D), each comprising seven items, for a total of 14 items. Responses are rated on a four-point scale ranging from 0 to 3. Positively worded items are scored as “not at all = 0,” “occasionally = 1,” “often = 2,” and “almost always = 3,” whereas negatively worded items are scored in reverse. Total scores for each subscale range from 0 to 21. According to widely used criteria in China, a subscale score of ≥8 indicates clinically significant anxiety or depressive symptoms. Psychometric evaluations have demonstrated that the HADS has good reliability [31], with Cronbach’s α coefficients ranging from 0.68 to 0.93 for the anxiety subscale and from 0.67 to 0.90 for the depression subscale, and test–retest correlation coefficients exceeding 0.80, supporting its applicability for emotional screening in non-psychiatric critically ill populations.

Participants completed the questionnaire independently based on their recent subjective experiences. For patients with mild cognitive impairment, trained researchers read each item aloud and documented patients’ verbal responses, thereby minimizing potential self-report bias.

### 2.4 Outcome measures

At 1 month after discharge, PTSD symptom severity was assessed using the Posttraumatic Stress Disorder Checklist for DSM-5 (PCL-5), which was administered to patients via email or WeChat. The PCL-5 was developed by Weathers et al. [11] and is aligned with the DSM-5 symptom framework for PTSD. It is widely used for symptom monitoring and screening in clinical research and follow-up settings. The scale comprises 20 items corresponding to four DSM-5 PTSD symptom clusters: cluster B (items 1–5), intrusive re-experiencing of trauma-related memories; cluster C (items 6–7), persistent avoidance of trauma-related stimuli, including activities, people, and objects; cluster D (items 8–14), negative alterations in cognition and mood; and cluster E (items 15–20), marked alterations in arousal and reactivity.

Each item is rated on a five-point scale from 0 to 4, reflecting the extent to which PTSD symptoms affected the respondent during the preceding month. The total score ranges from 0 to 80, with higher scores indicating greater symptom severity. A recommended screening cutoff in the range of 31–33 has been reported depending on the study population and screening objective, and scores within or above this range may indicate clinically significant posttraumatic stress symptoms [11]. The PCL-5 has demonstrated good psychometric properties [32], with a Cronbach’s α of 0.94 and test–retest reliability of 0.82.

### 2.5 Data preprocessing and missing data handling

Following completion of data collection, all records underwent unified cleaning and coding to address duplicate entries, logical contradictions, and inconsistencies. Ambiguous items were traced to their original sources for verification and subsequently standardized. After exclusion of extreme outliers, structurally invalid zero values, and records with unrecoverable missing data, a total of 500 valid observations were retained. These observations were first randomly divided into a development set (n=400) and an independent test set (n=100). The development set was then further split into a training set and a validation set at a ratio of 80%:20%. To prevent information leakage, all preprocessing procedures, including missing value handling and standardization of continuous variables, were fitted exclusively on the training set and then applied unchanged to the validation and test sets. The detailed data cleaning and standardization procedures are outlined below:

(1) Standardization of clinical indicator definitions

Data including time of emergency department presentation, time of transfer to the EICU, admission vital signs (e.g., heart rate, blood pressure, and oxygenation index), and records of emergency interventions (e.g., cardiopulmonary resuscitation) were extracted from the hospital electronic medical record system. Admission urgency was classified by trained research assistants according to predefined grading criteria based on electronic medical record data. Level 1 (extremely urgent): immediate life-support interventions were required (e.g., cardiopulmonary resuscitation or tracheal intubation), with transfer from the emergency department to the EICU within 30 minutes; Level 2 (urgent): risk of organ failure was present, requiring transfer to the EICU and initiation of monitoring within 1 hour; Level 3 (moderately urgent): the patient’s condition was relatively stable but required close observation, with transfer to the EICU within 2–4 hours; Level 4 (non-urgent): the condition was controllable, with transfer occurring after more than 4 hours or through planned admission (e.g., postoperative monitoring).

Delirium was assessed daily by nursing staff using the Confusion Assessment Method for the Intensive Care Unit (CAM-ICU) [33]. The presence of delirium on at least one day during the EICU stay was defined as “delirium present.” Sedation and agitation levels were evaluated every 4 hours using the Richmond Agitation–Sedation Scale (RASS). A day was classified as “coma” if all RASS scores recorded on that day were ≤ −3. The cumulative number of days with delirium and coma during the EICU stay was calculated. If delirium and coma occurred on the same day, that day was counted as a delirium day for the purpose of cumulative-day calculation. All variables were retrieved from the electronic medical record system according to predefined rules.

The Acute Physiology and Chronic Health Evaluation II (APACHE II) score was uniformly calculated using clinical data obtained within the first 24 hours after EICU admission.

Sedative medication exposure was defined as continuous intravenous infusion of benzodiazepines for at least 24 hours.

A history of mental illness was defined as a documented psychiatric diagnosis prior to EICU admission, excluding patients with severe psychiatric symptoms or those in an acute exacerbation phase.

Family visitation frequency and quality were evaluated based on healthcare staff observations and patients’ subjective reports. High-frequency, high-quality visitation was defined as at least one visit per day, lasting ≥30 minutes per visit, with provision of emotional support and active cooperation with medical staff communication. Low-frequency or low-quality visitation was defined as 2–3 visits per week, visits lasting <15 minutes, or visits characterized by anxiety that interfered with medical care. Absence of visitation was defined as no family visits due to factors such as distance or family conflict.

(2) Handling of duplicate and erroneous data

Duplicate records were identified using patients’ unique identifiers (e.g., hospitalization numbers) in combination with key variables, such as admission time and diagnostic information, and were subsequently removed. Erroneous data, including physiological measurements with values markedly outside clinically plausible ranges, were reviewed in consultation with clinicians and were corrected or excluded based on patients’ actual clinical conditions.

(3) Handling of missing values and outliers

A stratified strategy was applied for missing data handling. For numerical variables with a low proportion of missing values (<10%), median imputation was used. When the proportion of missing values was relatively high (≥10%), imputation was performed using the k-nearest neighbors (KNN) algorithm. Extreme outliers and structurally invalid zero values were identified during data cleaning and handled based on source verification and clinical plausibility. Records with unrecoverable missing data were excluded.

### 2.6 Feature selection and modeling strategy

Univariate analyses were first conducted to examine associations between candidate variables and PCL-5 scores, with the aim of preliminary feature screening and dimensionality reduction. For categorical predictors, group differences in PCL-5 scores were assessed using independent-samples t-tests or one-way analysis of variance, as appropriate. For continuous predictors, correlation analysis or univariate linear regression was used. A two-sided P < 0.05 was considered statistically significant. Within the development set, preliminary univariate screening was performed for variable reduction before model training. Based on statistical significance, together with considerations of clinical relevance and data completeness, 11 candidate variables were retained for subsequent model development. All preprocessing procedures requiring parameter estimation, including missing value imputation and standardization, were fitted exclusively on the training set and applied unchanged to the validation and test sets.

A CNN-Attention deep learning framework was subsequently employed to perform multi-output regression. The model input consisted of an 11-dimensional feature vector, while the output comprised five targets, including the total PCL-5 score and four symptom dimension scores. The model architecture incorporated one-dimensional convolutional layers to capture local feature patterns, along with channel attention and spatial attention modules to enhance the weighting and representation of salient features. In addition, the objective quantitative constraint that the total score equals the sum of the four sub-dimension scores was incorporated into the loss function to improve training efficiency and enhance consistency and interpretability of the predictions. EarlyStopping was applied during model training to mitigate overfitting.

This strategy reflected a prediction-oriented methodological trade-off, in which preservation of potentially informative variables was prioritized over additional regression-based feature reduction.

### 2.7 Model evaluation and comparison models

Model performance was evaluated using the coefficient of determination (R^2^), root mean square error (RMSE), mean absolute error (MAE), and mean absolute percentage error (MAPE). These metrics were further calculated on an independent test set to assess the generalization performance of the models. To evaluate the added value of the attention mechanism and the advantages of deep learning approaches, several comparison models were constructed, including multiple linear regression, backpropagation (BP) neural networks, and a CNN model without attention mechanisms. The pure CNN model shared the same structural parameters as the CNN-Attention model, with the attention modules removed. Although the model simultaneously predicted the total PCL-5 score and four symptom-cluster scores, prediction of the total PCL-5 score served as the primary basis for model comparison.

### 2.8 Reporting guideline

This study was reported in accordance with the Strengthening the Reporting of Observational Studies in Epidemiology (STROBE) statement for observational studies. The completed STROBE checklist is provided as S1 Checklist.

## 3 Current status of PTSD and influencing factors in mechanically ventilated EICU patients

### 3.1 Sample inclusion and baseline characteristics

During the study period, 652 questionnaires were distributed, and 500 valid observations were retained, yielding a valid response rate of 76.69%. After preprocessing, these observations were randomly divided into a development set (n = 400) and an independent test set (n = 100). Unless otherwise specified, the descriptive analyses reported in this section were based on the development set. The mean age of the 400 included patients was 38.03 ± 9.08 years, and males comprised approximately half of the cohort. Most patients were aged between 29 and 50 years (62.5%). Admission urgency was predominantly classified as level 1 or 2 (69.3%), indicating that the study population largely consisted of patients requiring highly urgent care, which is consistent with the clinical characteristics of the EICU setting. Invasive mechanical ventilation was the most common ventilation modality (52.5%), and 56.5% of patients received mechanical ventilation for ≤72 h. Approximately 20.0% of patients experienced delirium for a cumulative duration of ≥3 days. Additional baseline characteristics are summarized in S1 Table.

### 3.2 PTSD Symptom Burden and Distribution of PCL-5 Scores

(1) Distribution of PCL-5 scores and proportion above the screening cutoff:

At 1 month after discharge, the mean total PCL-5 score was 37.80 ± 9.10. For descriptive presentation, PCL-5 scores were grouped into four score ranges, and most scores fell within the range of 33–49 (60.8%). This finding indicates that a substantial proportion of patients continued to experience a significant PTSD symptom burden during the early post-discharge period. Patients with PCL-5 scores of 50–80 accounted for 10.0%, indicating a relatively high symptom burden in this subgroup. Although this proportion was small, these patients may warrant prioritized follow-up and targeted intervention. When a PCL-5 score of 33 was used as the reference screening cutoff, 283 patients met this criterion, representing 70.8% of the development sample (Table 1).

**Table 1:** Distribution of PCL-5 score ranges in mechanically ventilated young and middle-aged EICU patients.

Score range	Score range group	Score(M ± SD)	Frequency (n)	Percentage (%)
0 ～ 19	lower symptom burden	18.00 ± 0.00	3	0.8
20 ～ 32	moderate symptom burden	29.14 ± 0.63	114	28.5
33 ～ 49	elevated symptom burden	39.78 ± 0.48	243	60.8
50 ～ 80	high symptom burden	57.31 ± 1.63	40	10.0

(2) Dimension scores:

Based on the comparison of mean item scores across dimensions, the avoidance dimension exhibited the highest mean item score (2.00 ± 0.70). This was followed by alterations in arousal and reactivity (1.95 ± 0.60), negative alterations in cognition and mood (1.87 ± 0.59), and intrusion symptoms (1.84 ± 0.60). These findings indicate that avoidance-related symptoms were more pronounced in this population (Table 2).

**Table 2:** Scores of PTSD symptom dimensions (n = 400).

Dimension	Score Range	Dimension Score(M ± SD)	Number of Items	Mean Item Score (M ± SD)
Intrusion symptoms (B)	0 ～ 20	9.2 ± 3.0	5	1.84 ± 0.60
Avoidance symptoms (C)	0 ～ 8	4.0 ± 1.4	2	2.00 ± 0.70
Negative alterations in cognition and mood (D)	0 ～ 28	13.1 ± 4.1	7	1.87 ± 0.59
Alterations in Arousal and Reactivity (E)	0 ～ 24	11.7 ± 3.6	6	1.95 ± 0.60
Total score	0 ～ 80	37.80 ± 9.10	20	1.90 ± 0.46

Note: Scores are derived from the PCL-5. Dimensions B-E correspond to the four symptom clusters of PTSD.

### 3.3 Candidate variables identified by univariate analysis

#### 3.3.1 Results of univariate analysis.

Independent-samples t-tests and one-way analysis of variance were applied to examine factors associated with PCL-5 scores. A total of 11 candidate variables showed statistical significance (P < 0.05) and were retained for subsequent model development (Table 3).

**Table 3:** Comparison of PCL-5 scores across categories of the 11 Candidate variables (M ± SD).

Factor	Category	Frequency (n)	PCL-5 Score (M ± SD)	F/t Value	P Value
Age	18 ~ 28	42	40.2 ± 8.8	4.21	0.016
	29 ~ 50	248	38.6 ± 9.1		
	51 ~ 59	110	35.9 ± 8.7		
Family visitation frequency and quality	High frequency, high quality	178	35.8 ± 8.6	18.37	<0.001
	Low frequency or low quality	152	39.3 ± 8.9		
	No visitation	70	42.7 ± 9.4		
History of mental illness	Yes	33	45.1 ± 8.7	9.98	0.002
	No	367	37.5 ± 9.0		
Admission urgency	Level 1 (extreme urgency)	123	42.5 ± 9.2	16.64	<0.001
	Level 2 (high urgency)	154	38.9 ± 8.8		
	Level 3 (moderate urgency)	85	35.7 ± 9.1		
	Level 4 (non-urgent)	38	34.0 ± 8.2		
Length of EICU stay (days)	≤5	132	35.2 ± 8.3	12.08	0.007
	6 ~ 10	140	38.7 ± 9.0		
	＞10	128	41.3 ± 8.5		
Cumulative days of delirium (CAM-ICU +)	0	170	35.6 ± 8.4	22.15	<0.001
	1 ~ 2	150	39.8 ± 8.8		
	≥3	80	43.3 ± 9.6		
Mode of mechanical ventilation	Non-invasive ventilation	120	36.0 ± 8.5	17.02	<0.001
	Invasive ventilation	210	39.1 ± 9.0		
	Both modes	70	41.8 ± 9.4		
Duration of mechanical ventilation	≤72h	226	36.5 ± 8.6	8.56	0.012
	＞72h	174	40.5 ± 9.2		
Received CRRT/ ECMO therapy	Yes	112	43.0 ± 9.0	4.01	0.041
	No	288	37.2 ± 8.9		
Anxiety positive (HADS-A ≥ 8)	Yes	295	42.4 ± 8.3	19.72	<0.001
	No	105	34.7 ± 8.2		
Depression positive (HADS-D ≥ 8)	Yes	267	41.8 ± 8.5	6.83	0.023
	No	133	35.3 ± 8.6		

#### 3.3.2 Analysis of significant factors.

(1) Age:
Significant differences in PCL-5 scores were observed across age groups (P < 0.05). Higher scores were found in patients aged 18–28 years. This result differs from previous findings on age-related PTSD risk. Some studies have     reported a higher susceptibility among younger patients, whereas others have suggested increased risk in individuals aged > 45 years or no clear association with age. Such inconsistencies may be explained by differences in family responsibilities, coping strategies, and trauma-related cognitive processing.
(2) Family visitation frequency and quality (social support):
 Lower PCL-5 scores were generally observed in patients receiving high-frequency and high-quality family visitation. In contrast, higher scores were found in patients with no visitation or low-quality visitation (P < 0.05). Social support is widely regarded as a key protective factor in both the onset and alleviation of PTSD. It may mitigate traumatic experiences by providing emotional reassurance, clarifying information, and enhancing perceived safety.
(3) History of mental illness:
 Higher PCL-5 scores were observed in patients with a history of mental illness (P < 0.05). Pre-existing psychiatric conditions may reduce stress tolerance and impair psychological regulation. As a result, patients may be more vulnerable to sustained traumatic responses when exposed to critical illness and invasive medical procedures.
(4) Admission urgency:
 Greater admission urgency, such as Level 1–2 classification, was associated with higher PCL-5 scores. Urgent admissions are often characterized by sudden onset, intense loss of control, and frequent invasive interventions. These experiences may intensify subjective trauma and increase cumulative PTSD risk.
(5) Length of EICU stay:
 Longer EICU stays were associated with higher PCL-5 scores (P < 0.05). Extended hospitalization often reflects greater disease severity and prolonged treatment exposure. During this period, patients are repeatedly exposed to pain, sleep disruption, and restricted communication in an unfamiliar environment. These factors may facilitate the formation of negative ICU memories and prolong stress-related responses.
(6) Cumulative days of delirium:
 Higher PCL-5 scores were observed with increasing cumulative days of delirium (P < 0.05). Delirium may result in fragmented and negatively valenced memories, and in some cases, delusional memory content. These memory disturbances can give rise to recurrent intrusive recollections. Delirium has been identified in multiple studies as a major risk pathway for ICU-related PTSD.
(7) Mode of mechanical ventilation:
 PCL-5 scores differed significantly across mechanical ventilation modes (P < 0.05). Ventilation strategies characterized by greater invasiveness and stronger physical restriction were generally associated with increased experiences of fear and helplessness. These findings highlight the contribution of the subjective traumatic experience of medical treatment to PTSD development.
(8) Duration of mechanical ventilation (≤ 72 h vs > 72 h):
 Longer durations of mechanical ventilation were associated with higher PCL-5 scores (P < 0.05). Previous studies have shown that PTSD risk increases markedly when ventilation exceeds 72 hours. Prolonged ventilation is also linked to a higher incidence of delirium and greater exposure to sedative agents. These factors may exert cumulative effects on PTSD risk.
(9) Receipt of CRRT/ ECMO therapy:
 Higher PCL-5 scores were observed in patients who received CRRT/ ECMO therapy (P < 0.05). This variable likely reflects increased disease severity and more intensive exposure to invasive medical procedures, thereby amplifying traumatic experiences. These findings suggest that this subgroup should be considered a priority population for psychological risk monitoring and intervention.
(10) Anxiety positive (HADS-A ≥ 8) and depression positive (HADS-D ≥ 8):
 Patients screening positive for anxiety exhibited significantly higher PCL-5 scores (P < 0.05). Acute psychological stress is regarded as one of the strongest and most modifiable risk factors for PTSD. Anxiety may contribute to symptom persistence by enhancing threat perception and reinforcing avoidance behaviors. Patients screening positive for depression also demonstrated higher PCL-5 scores (P < 0.05). PTSD frequently co-occurs with anxiety and depression, with substantial symptom overlap. Such comorbidity may further aggravate social functional impairment and reduced quality of life. These findings indicate that a comprehensive assessment strategy, rather than single-symptom screening, should be adopted in clinical practice.

### 3.4 Rationale for excluding multivariate analysis in model construction

In this study, multivariable regression was not used as an additional variable-selection step before model construction. First, the primary aim was prediction rather than estimation of independent effect sizes for individual variables. Candidate variables were therefore retained on the basis of univariate screening, clinical relevance, and data completeness, so as to preserve potentially informative predictors for subsequent model development. Second, the planned CNN-Attention model was intended to capture complex and potentially nonlinear relationships among predictors and outcomes. Under this prediction-oriented framework, further variable reduction based solely on conventional multivariable regression was not considered necessary. Third, a multiple linear regression model was included as one of the benchmark models for comparative evaluation, allowing assessment of the added value of the deep learning approach relative to a conventional statistical model.

Accordingly, based on the candidate variables identified through univariate analysis, a deep learning approach was used for subsequent prediction modeling in this study. This strategy was consistent with the prediction-oriented objective of the study and the anticipated complexity of the relationships among variables. SHAP Kernel Explainer was subsequently applied to improve interpretation of the final model.

## 4 Construction and validation of a CNN-attention-based PCL-5 prediction model

### 4.1 Construction of the CNN-attention neural network model

A CNN-Attention multi-output regression model was developed in this study to predict the total PCL-5 score and the scores of four symptom dimensions at 1 month after discharge in young and middle-aged mechanically ventilated patients in the EICU. Eleven feature variables identified through univariate analysis, together with considerations of clinical relevance and data completeness, were used as model inputs. Five continuous outcomes were defined as outputs, including the total PCL-5 score and four dimension-specific scores. Compared with conventional regression-based approaches, the present prediction task involves multiple demographic, clinical, treatment-related, and psychological variables that may interact in complex and potentially nonlinear ways. Under such circumstances, traditional linear models may have limited ability to capture higher-order feature interactions and latent structural patterns in the data. Therefore, a CNN-Attention framework was adopted in this study to improve the representation of structured clinical features and to enhance prediction performance for multidimensional PTSD symptom outcomes. To accommodate structured clinical data, the 11 features were arranged in a fixed order and organized into a one-dimensional sequence. This sequence was then fed into a 1D-CNN to capture local interaction patterns between adjacent features. An attention mechanism was subsequently incorporated to enhance the model’s focus on informative features.

This design was based on the assumption that, when ordered according to a predefined clinical logic, neighboring variables may contain informative local dependencies that can be learned through convolutional operations. In this way, the model was expected to extract higher-level and more representative latent features from the original 11-dimensional input vector. The dataset comprised 400 samples. Each sample included 11 feature variables as independent variables and five target variables as dependent variables. These inputs can be represented as an 11-dimensional feature vector. Given the ability of convolutional neural networks to model local patterns and structured feature relationships, a one-dimensional convolutional architecture was considered suitable for this non-image learning task.

While conventional CNNs have shown effectiveness in sequence modeling and structured feature extraction, their ability to capture global dependencies remains limited. To address this limitation, a dual attention mechanism was integrated into the CNN framework in this study. This mechanism consisted of channel attention and spatial attention modules. The purpose of introducing this mechanism was not only to improve predictive performance by emphasizing informative features, but also to facilitate interpretation of which feature dimensions and positions contributed more strongly to the prediction results.

**Fig 1:**
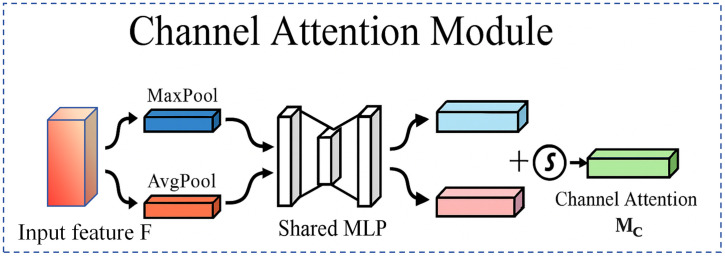
Logical framework of the channel attention mechanism.

The channel attention mechanism is illustrated in Fig 1. This module was designed to learn the relative importance of each feature channel and to assign adaptive weights accordingly. Channel-wise statistical information was obtained using global average pooling (GAP) and global max pooling (GMP). Nonlinear feature relationships were then modeled using a shared multilayer perceptron (MLP) to generate channel attention weights. The original feature maps were subsequently recalibrated through channel-wise weighting, allowing important channels to be emphasized while redundant information was suppressed.

The underlying mathematical formulation is described as follows:


$$\begin{array}{c} {\text{M}}_{\text{c}}=\sigma \left(\text{MLP}\left(\text{AvgPool}(\text{I})\right)+\text{MLP}\left(\text{MaxPool}(\text{I})\right)\right) \end{array}$$ (1)

$$\begin{array}{c} {\text{F}}^{\prime }={\text{M}}_{\text{c}}⊙\text{F} \end{array}$$ (2)

Here, F denotes the input feature, Mc represents the channel attention weight, “⊙” indicates element-wise multiplication along the channel dimension, and σ denotes the sigmoid function.

**Fig 2:**
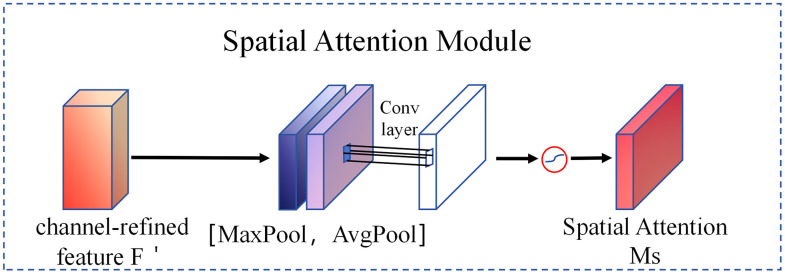
Logical framework of the spatial attention mechanism.


The logical framework of the spatial attention mechanism is illustrated in Fig 2. The spatial attention module is designed to identify important spatial locations within the feature representation. Average pooling and max pooling are applied along the channel dimension to extract spatial descriptors. A convolution operation is then used to generate a spatial attention map, which enhances feature responses at salient positions. The underlying mathematical formulation is given as follows:

$$\begin{array}{c} {\text{M}}_{\text{s}}=\sigma \left(\text{Conv1d}\left(\left[\text{AvgPool}(\text{F});\text{MaxPool}(\text{F})\right]\right)\right) \end{array}$$ (3)

$$\begin{array}{c} {\text{F}}^{\prime }={\text{M}}_{\text{s}}⊙\text{F} \end{array}$$ (4)

Here, F denotes the input feature, M s denotes the generated spatial attention weight, σ denotes the sigmoid function, and “⊙” indicates element-wise weighting across spatial positions.

To enable CNN-based extraction of local patterns, the input vector x ∈ R^11^ is treated as a single-channel sequence:


$$\begin{array}{c} \text{X}\in {\text{R}}^{\text{1}\times \text{1}\text{1}} \end{array}$$ (5)

A one-dimensional convolutional layer is employed for feature extraction:


$$\begin{array}{c} {\text{F}}_{\text{1}}=\sigma \left({\text{W}}_{\text{1}}*\text{X}+{\text{b}}_{\text{1}}\right) \end{array}$$ (6)

Where ${\text{W}}_{\text{1}}\in {\text{R}}^{\text{C}\times \text{k}}$ denotes the convolution kernel, C denotes the number of output channels, which is configured to increase from 16 to 32, k denotes the kernel size and is set to 3, σ denotes the sigmoid activation function. The output feature map ${\text{F}}_{\text{1}}\in {\text{R}}^{\text{C}\times \text{L}}$, where L = 11 (padding = 1).

**Fig 3:**
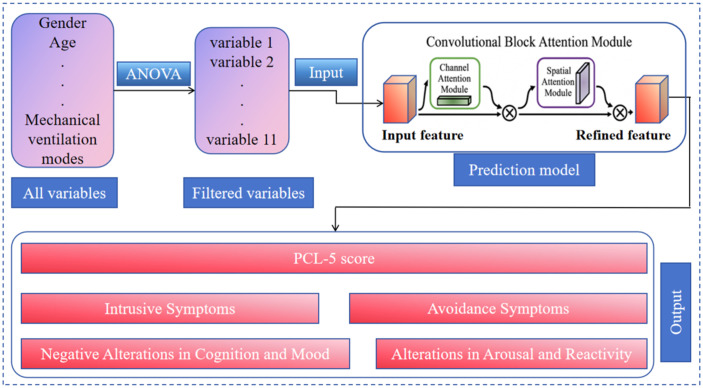
Overall workflow of the prediction model.

The overall architecture of the prediction model is illustrated in Fig 3. The attention module is inserted after the second convolutional block (C = 32). This design allows the convolutional layers to first extract local feature representations and then enables the attention mechanism to adaptively reweight these learned representations, thereby improving both feature discrimination and model robustness.

The main modules of the neural network model and their corresponding parameters are summarized in Table 4. As CNNs were applied to a non-image learning task in this study, only one-dimensional convolutional layers were employed.

**Table 4:** Neural network modules and parameter settings.

Module	Value
Input dimension	11
Output dimension	5
Conv1d layer (channels)	16
Convolution kernel size	3
Dropout rate	0.3
Channel attention MLP	16 → 8 → 16
Spatial attention convolution kernel	3
Optimizer	Adam
Learning rate	0.001
Batch size	16
Training epochs	1000 (with EarlyStopping of 100 patience)

Overall, the CNN-Attention model in this study was designed to leverage the local feature extraction capability of 1D-CNN and the adaptive feature-weighting capacity of dual attention mechanisms for the prediction of multidimensional PTSD symptom severity. By jointly predicting the total PCL-5 score and four symptom-cluster scores, the model was intended to provide not only an overall estimate of symptom burden but also a more fine-grained symptom profile to support early risk stratification and follow-up planning.

### 4.2 Loss function

The input sample is denoted as x ∈ *R*^11^, and the corresponding output is y ∈ R^5^. The training dataset consists of N = 400 samples. The objective is to learn a mapping function *f*_*θ*_: *R*^11^ → *R*^5^, such that the predicted output ${\hat{\text{y}}=\text{f}}_{\theta }(\text{x})$ closely matches the ground truth y.

The mean squared error (MSE) was adopted as the primary loss function and is defined as follows:


$$\begin{array}{c} {\text{L}}_{\text{MSE}}=\frac{\text{1}}{\text{N}}\sum_{\text{i}=}^{\text{N}}\overset{\text{2}}{\underset{\text{2}}{\left\|{\hat{\text{y}}}^{\left(\text{i}\right)}={\text{y}}^{\left(\text{i}\right)}\right\|}} \end{array}$$ (7)

Where ${\hat{\text{y}}}^{\left(\text{i}\right)}$ is predicted value, ${\text{y}}^{\left(\text{i}\right)}$ is actual value.

In this study, the relationship among the five variables Y, y_1_, y_2_, y_3_, and y_4_ is defined as Y = y_1_ + y_2_ + y_3_ + y_4_. This relationship was introduced into the loss function in the form of a dimensionless constraint term. This design was intended to improve the internal consistency of the model outputs, reduce the risk of excessive discrepancy between the predicted total score and the sum of the predicted dimension-specific scores, and thereby enhance the interpretability of the prediction results. The constraint function is formulated as follows:


$$\begin{array}{c} {\text{L}}_{\text{constraint}}=\frac{\text{1}}{\text{N}}\sum_{\text{i}=}^{\text{N}}{\left(\frac{{\hat{\text{Y}}}^{\left(\text{i}\right)}-\sum_{\text{j}=}^{\text{4}}\overset{\left(\text{i}\right)}{\underset{\text{j}}{\hat{\text{y}}}}}{{\hat{\text{Y}}}^{\left(\text{i}\right)}+\varepsilon }\right)}^{\text{2}} \end{array}$$ (8)

Here, $\hat{\text{Y}}$ denotes the predicted value of Y (the total score), and $\overset{\left(\text{i}\right)}{\underset{\text{j}}{\hat{\text{y}}}}$ denotes the predicted value of each dimension-specific subscore. The parameter ε is a small positive constant (10^−6^), introduced to avoid division by zero.

The overall loss function is defined as a weighted combination of the primary loss term and the constraint term:


$$\begin{array}{c} {\text{L}}_{\text{total}}={\text{L}}_{\text{MSE}}+\lambda \cdot {\text{L}}_{\text{constraint}} \end{array}$$ (9)

Here, λ represents the weighting coefficient and was set to 0.1. By introducing this auxiliary constraint, the model was encouraged not only to minimize prediction error for each output variable, but also to maintain coherence between the predicted total score and the predicted symptom-dimension scores.

### 4.3 Prediction model error analysis

**Fig 4:**
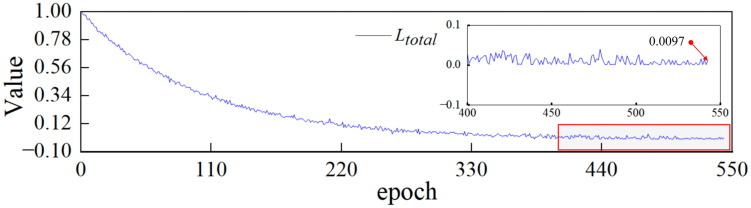
Loss decrease curve.

Among the 400 samples used for model development, the dataset was split into a training set and a validation set using an 80%/ 20% ratio. An EarlyStopping strategy was applied during training. Model training was terminated when the validation loss failed to decrease over multiple consecutive epochs, thereby preventing overfitting to the training data. The training loss convergence curve is presented in Fig 4. Early stopping was triggered at approximately 542 epochs, with a final loss of approximately 0.0097. As illustrated in the figure, the incorporation of an explicit constraint resulted in a smooth loss reduction throughout the training process, without noticeable oscillations.

**Fig 5:**
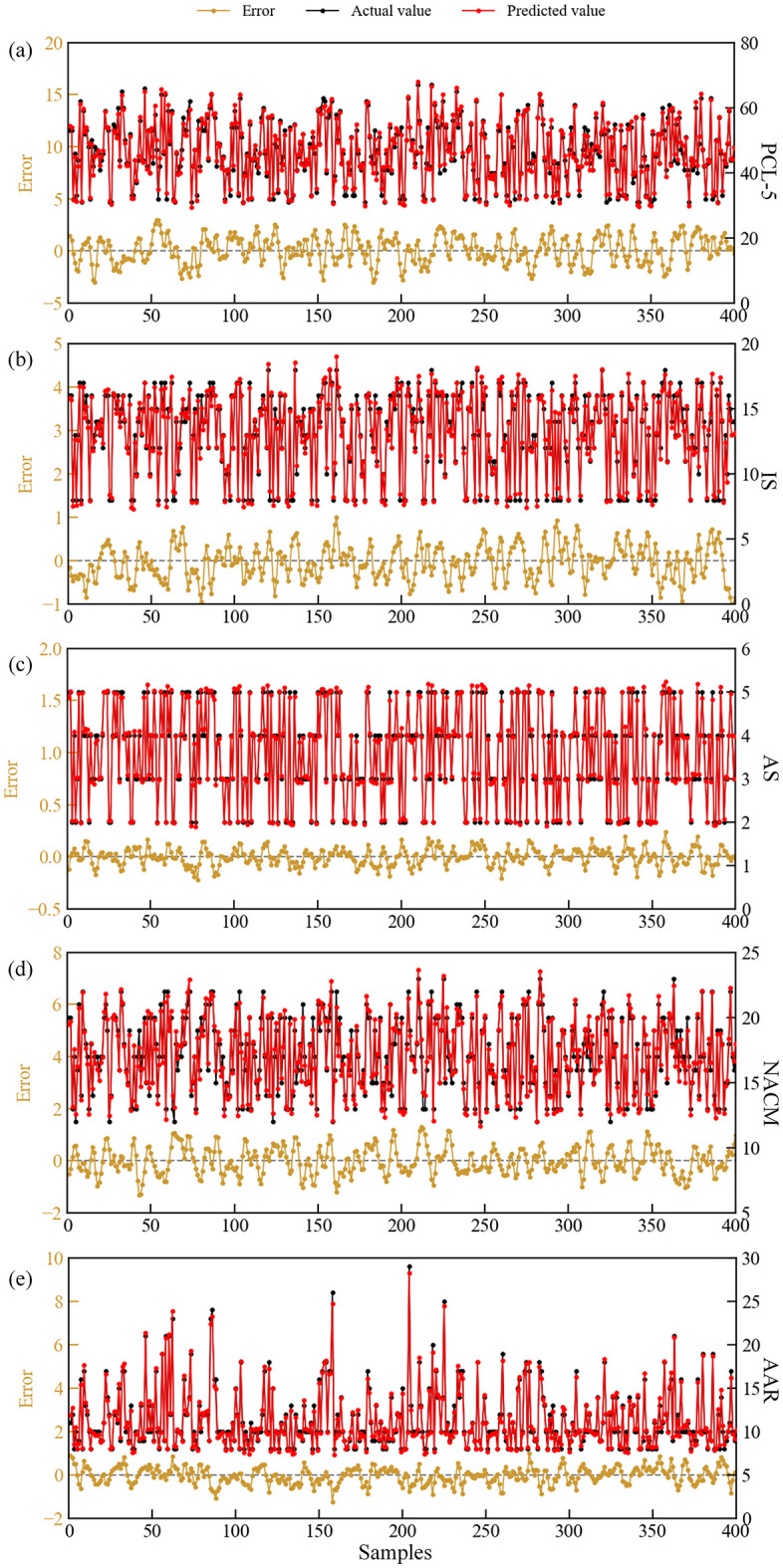
Comparison of true and predicted values for each score.

The outcome variables in this study were continuous-valued scores with integer scales. As the task is inherently a regression problem, the data were not treated as discrete integer points. Predicted values were therefore retained as decimal numbers and compared with the corresponding ground truth values. The comparison curves of true and predicted scores for both the training set and the validation set are presented in Fig 5. The horizontal axis labeled “Samples” represents individual sample indices only and does not imply any relational ordering among individuals. As shown in the figure, the discrepancies between predicted and true values for all five scores were small, and no outliers were observed. These results suggest that the model achieved good fitting performance in both the training and validation sets, with no obvious evidence of overfitting during model development. Overall, the CNN-Attention model demonstrated high predictive accuracy for both the total PCL-5 score and the four symptom dimension scores.

Multiple error metrics, including R^2^, RMSE, MAE, and MAPE, were applied to evaluate the performance of the proposed prediction model, as summarized in Table 5. The CNN-Attention model demonstrated strong predictive performance for PTSD symptoms. All dimensions achieved R^2^ values greater than 0.94. Among them, the Alterations in Arousal and Reactivity dimension exhibited the best goodness of fit, with an R^2^ of 0.9917. The overall mean R^2^ value reached 0.9799, which exceeded the conventional benchmark of 0.7 commonly adopted in psychological research.

**Table 5:** Multiple error evaluation metrics for prediction results.

Items	R^2^	RMSE	MAPE	MAE
PCL-5 score	0.979733	1.363818	2.318040%	1.090000
Intrusive Symptoms	0.979146	0.450000	1.329229%	0.202500
Avoidance Symptoms	0.98100	0.091331	1.123450%	0.0734450
Negative Alterations in Cognition and Mood	0.949156	0.630476	2.323535%	0.397500
Alterations in Arousal and Reactivity	0.991685	0.316228	0.662622%	0.100000

Comparative analysis across symptom dimensions showed that the Alterations in Arousal and Reactivity dimension achieved the most favorable performance, with R^2^ = 0.9917, RMSE = 0.3162, MAPE = 0.66%, and MAE = 0.1. The Negative Alterations in Cognition and Mood dimension demonstrated relatively lower performance (R^2^ = 0.9492). The largest prediction errors were observed for the total PCL-5 score (RMSE = 1.3638, MAE = 1.09), although the corresponding R^2^ value remained high at 0.9797.

Analysis of consistency across error metrics indicated a high degree of agreement among the four evaluation indices for all dimensions. For the Alterations in Arousal and Reactivity dimension, the discrepancy between RMSE (0.316) and MAE (0.1) suggested a positively skewed error distribution. The MAPE for the total PCL-5 score (2.32%) was notably higher than that of the other dimensions, reflecting larger absolute prediction errors. This effect is likely attributable to the cumulative nature of the total PCL-5 score, which is derived from the sum of four subscale scores. Despite the inclusion of a constraint-based loss term, error accumulation remained unavoidable.

From a practical perspective, the prediction errors for the four symptom dimensions were relatively small, with MAE values ranging from 0.07 to 0.40. The MAE for the total PCL-5 score was 1.09, which remained low relative to the overall scale range. These findings suggest that the proposed model achieved a high level of prediction accuracy, particularly for the symptom-dimension outputs.

Taken together, results from multiple evaluation metrics indicate that the CNN-Attention model effectively captures the nonlinear relationships among PTSD symptom dimensions. The model achieved clinically acceptable prediction accuracy, particularly at the symptom-dimension level, and may provide methodological support for future digital mental health assessment and early risk stratification.

### 4.4 Validation of the generalization ability of the prediction model

**Fig 6:**
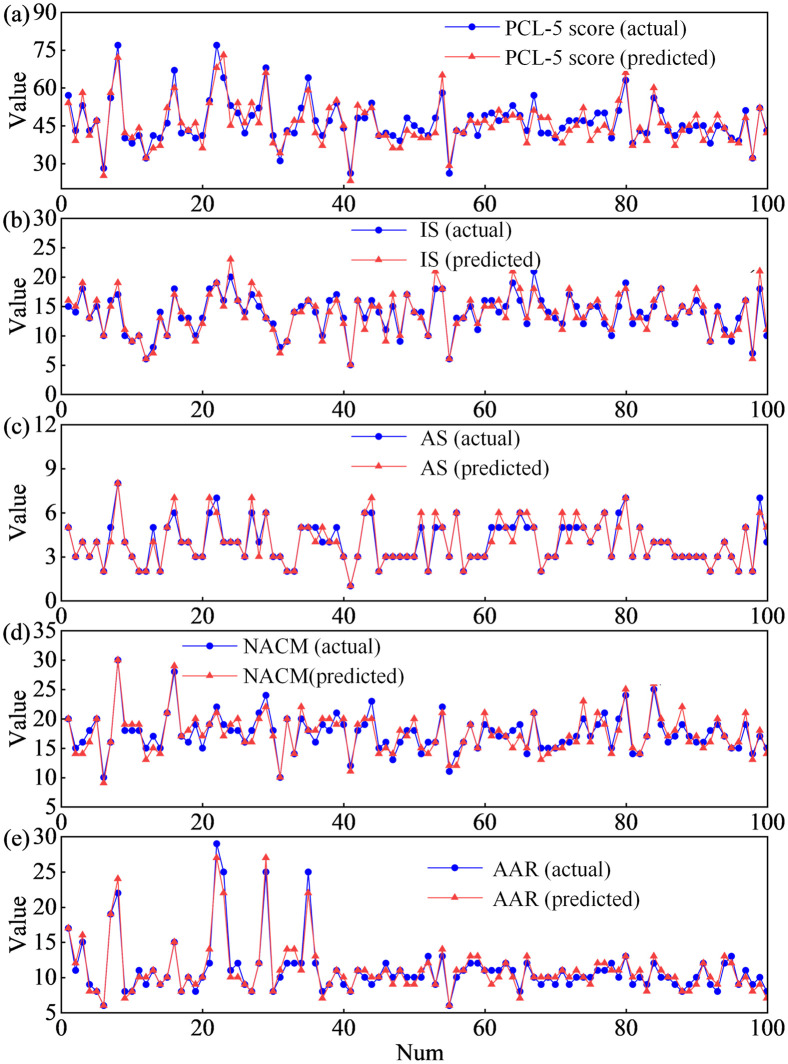
Comparison of true and predicted values for each score in the independent test set.

Although the CNN-Attention neural network achieved high predictive accuracy on the training and validation datasets, its generalization performance required further evaluation using unseen data. Therefore, the model was further evaluated using an independent test set consisting of 100 samples that were not included in the training or validation process. The comparison between true and predicted values for the independent test set is presented in Fig 6. Compared with the results shown in Fig 5, a moderate reduction in prediction accuracy was observed across all symptom dimensions. Nevertheless, the trained CNN-Attention prediction model maintained a high level of accuracy on the independent test set, indicating satisfactory generalization performance.

Multiple error metrics were applied to evaluate the predictive accuracy of the proposed model on the independent test dataset, as summarized in Table 6. Among the five outputs, the highest prediction accuracy was achieved for Avoidance Symptoms, with an R^2^ of 0.8844 and the lowest error values across several evaluation metrics. In particular, the Alterations in Arousal and Reactivity dimension achieved the highest goodness of fit (R^2^ = 0.9286)., while Avoidance Symptoms showed the lowest absolute prediction errors. In contrast, prediction performance for the total PCL-5 score was relatively lower, with an R^2^ of 0.804 and the highest error values. The MAPE for the total PCL-5 score reached 7.43%, which was notably higher than that of other dimensions, indicating larger absolute prediction errors. This result is attributable to the cumulative nature of the total PCL-5 score, which is derived from the sum of four subscale scores. Although a constraint-based loss function was applied to enforce consistency between the total score and the subscale scores, error accumulation remained unavoidable. Overall, all predicted scores achieved R^2^ values exceeding 0.8, surpassing the conventional threshold of 0.7 commonly adopted in psychological research. These findings suggest that the CNN-Attention model exhibited acceptable robustness and generalization ability in the independent test set.

**Table 6:** Multiple error evaluation metrics in the independent test set.

Items	R^2^	RMSE	MAPE	MAE
PCL-5 score	0.803523	2.163964	7.426456%	1.935465
Intrusive Symptoms	0.853026	1.236932	6.957867%	0.970000
Avoidance Symptoms	0.884370	0.479583	4.469048%	0.230000
Negative Alterations in Cognition and Mood	0.816830	1.367479	6.404923%	1.110000
Alterations in Arousal and Reactivity	0.928620	1.019804	6.697513%	0.760000

### 4.5 SHAP analysis

To enhance model interpretability, the SHAP Kernel Explainer was applied to interpret the final prediction model. As Kernel SHAP requires a predefined baseline distribution, a background set was constructed by randomly sampling from the model development dataset. SHAP values for each feature were then computed on the independent test set for both the total score and the dimension-specific scores. This procedure enabled the identification of global feature importance rankings and corresponding effect directions. It should be noted that SHAP explanations quantify feature contributions within the model and do not imply causal relationships.

SHAP interpretation depends on a baseline distribution (background) to approximate the expected model output for the target population. In this study, missing value imputation and feature normalization were completed during the training phase. All preprocessing parameters were estimated exclusively from the training data and subsequently applied to the validation and test datasets to prevent information leakage. Accordingly, SHAP calculations were conducted on feature matrices that were preprocessed in an identical manner to the model inputs.

Random samples from the model development data (training set) were selected to construct the background set. To avoid baseline instability caused by an insufficient background size while maintaining computational feasibility for Kernel SHAP, K = 100 samples were chosen. Stratified sampling was performed based on PCL-5 scores using a four-level stratification strategy to ensure coverage across different levels of symptom burden. SHAP values were then computed on the independent test set to evaluate the interpretability performance of the model on extrapolated data.

**Fig 7:**
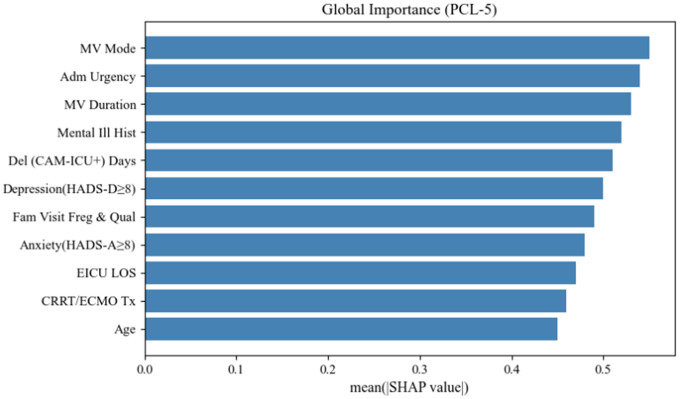
Global importance bar plot for the total PCL-5 score based on mean (|SHAP|).

Fig 7 presents the global feature importance for predicting the total PCL-5 score based on SHAP analysis. According to the mean(|SHAP|) values, mechanical ventilation mode (MV Mode), admission urgency (Adm Urgency), duration of mechanical ventilation (MV Duration), history of mental illness (Mental Ill Hist), and cumulative days of delirium positivity (Del (CAM-ICU+) Days) emerged as the most influential predictors, exerting the greatest impact on model output. Depressive symptoms (Depression (HADS-D ≥ 8)) and anxiety symptoms (Anxiety (HADS-A ≥ 8)) also demonstrated substantial contributions, indicating that affective symptoms play an important role in PTSD risk prediction. By comparison, age (Age) and CRRT/ ECMO treatment (CRRT/ECMO therapy) showed relatively smaller contributions to the prediction of the total PCL-5 score.

Collectively, these findings underscore the importance of ICU-related clinical factors, including mechanical ventilation characteristics, psychiatric history, and delirium burden, in PTSD risk assessment. They further suggest that disease severity and mental health–related symptoms represent core determinants of PTSD risk in this patient population.

**Fig 8:**
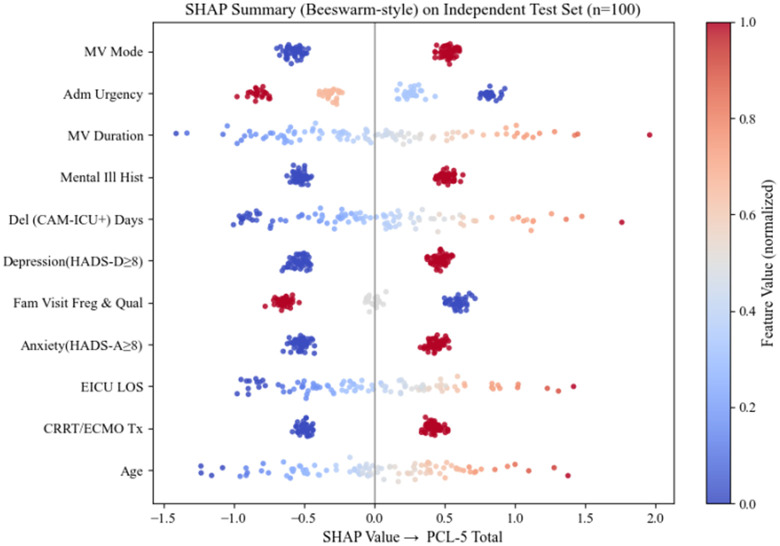
SHAP Summary plot for the total PCL-5 score.

Fig 8 further illustrates the directionality of feature contributions to the prediction of the total PCL-5 score and the relationships between feature values and their corresponding SHAP values. The SHAP summary plot shows that mechanical ventilation mode (MV Mode) and duration of mechanical ventilation (MV Duration) are associated with predominantly positive SHAP values, indicating a positive contribution to the total PCL-5 score. These results suggest that longer ventilation duration and more invasive ventilation strategies are linked to increased PTSD risk. Similarly, cumulative days of delirium positivity (Del (CAM-ICU+) Days) and length of ICU stay (EICU LOS) exhibited predominantly positive SHAP values, further supporting the association between greater illness severity and elevated PTSD risk.

By contrast, family visitation frequency and quality (Fam Visit Freq & Qual) demonstrated a substantial negative contribution to the total PCL-5 score, as reflected by left-shifted SHAP values. This finding suggests a potential protective effect of higher-quality family support in mitigating PTSD risk. Moreover, the positive SHAP values associated with depressive symptoms (Depression (HADS-D ≥ 8)) and anxiety symptoms (Anxiety (HADS-A ≥ 8)) underscore the importance of emotional distress in PTSD risk evaluation.

Overall, these findings indicate that ICU-related clinical factors and mental health conditions play a central role in PTSD risk prediction, while also highlighting the potential protective influence of family support in this patient population.

### 4.6 Performance comparison across multiple models

The PCL-5 score represents the total score and is defined as the sum of the remaining four dimension scores. As a mathematical relationship among output variables was incorporated into the loss function as a constraint term, the prediction error of the PCL-5 score reflects the accumulated errors of the four dimension-specific outputs. Using the PCL-5 score as the primary reference outcome, the predictive performance of the CNN-Attention model was compared with that of conventional models, including multiple linear regression, BP, and CNN, as summarized in Table 7. For the CNN model, all parameters were kept identical to those of the CNN-Attention model, except for the absence of the attention mechanism.

**Table 7:** Prediction errors of different models for the PCL-5 score.

Items	R^2^	RMSE	MAPE	MAE
CNN-Attention	0.979733	1.363818	2.318040%	1.090000
Multiple linear regression	0.702753	2.964245	9.356743%	2.636234
BP	0.784370	1.773646	6.736567%	2.300001
CNN	0.827465	1.497254	5.577453%	1.718366
Random Forest	0.909753	1.653350	3.083632%	1.409785
XGBoost	0.887608	1.570011	4.191103%	1.507732

The results show that the multivariate linear regression model exhibited the weakest fitting performance for high-dimensional data. In addition, compared with the CNN model without attention, the CNN-Attention model achieved markedly improved performance across all error metrics. These findings suggest that the incorporation of an attention mechanism substantially enhances the feature extraction capability of CNNs for high-dimensional, small-sample data. The CNN-Attention model is therefore well suited for predicting PCL-5 scores in mechanically ventilated patients in the EICU.

In summary, the 1D CNN-Attention model proposed in this study makes effective use of the strong feature extraction capability of convolutional neural networks. To better suit the data type used in this study, one-dimensional convolution kernels were adopted and an attention mechanism was introduced. Compared with the original CNN and other commonly used models, the proposed model demonstrated superior performance. Although there was a slight decline in performance on the held-out independent test set, the model still remained within an acceptable usable range. This model can provide some reference value for clinical diagnosis.

### 4.7 Hardware and software environment and computational cost

To enhance reproducibility and facilitate benchmarking, the hardware and software environment used for model development is described. All experiments were conducted on a desktop workstation equipped with an Intel Core i7-10700 CPU, 32 GB RAM, and an NVIDIA GeForce RTX 2060 GPU with 6 GB of memory. The operating system was Windows 10 64-bit. The models were implemented using Python 3.9.13. Neural network models were developed using PyTorch 2.0.1 with CUDA 11.8, while conventional machine learning models were implemented using scikit-learn 1.2.2 and XGBoost 1.7.6.

For fair comparison, all models were trained and evaluated using the same development and independent test datasets. The neural network models were trained with a batch size of 16. For the CNN-Attention model, the maximum number of epochs was set to 1000, with EarlyStopping patience set to 100. In the final training process, early stopping was triggered at approximately 542 epochs. The approximate computational cost of each model is summarized in Table 8.

**Table 8:** Approximate computational cost of different models.

Model	Implementation environment	Approximate training time	Inference time on test set
Multiple linear regression	CPU, scikit-learn	<1 s	<0.1 s
Random Forest	CPU, scikit-learn	9 s	0.2 s
XGBoost	CPU, XGBoost	12 s	0.1 s
BP neural network	GPU, PyTorch	39 s	<0.1 s
CNN without attention	GPU, PyTorch	54 s	<0.1 s
CNN-Attention	GPU, PyTorch	1 min 18 s	<0.1 s
CNN-Attention + SHAP interpretation	GPU/CPU, PyTorch + SHAP	6 min 40 s	/

The CNN-Attention model required a slightly longer training time than the BP neural network and the CNN model without attention because of the additional channel and spatial attention modules. Nevertheless, the complete workflow, including model training, prediction on the independent test set, and SHAP-based interpretation, was completed within 10 minutes using a commonly available RTX 2060 GPU workstation. These results indicate that the proposed model does not require high-performance computing infrastructure and can be reproduced using standard desktop-level hardware.

## 5 Discussion

### 5.1 Principal findings

This prospective cohort study developed and validated a CNN-Attention multi-output regression model to predict post-discharge PTSD symptom severity (PCL-5 total score and four symptom-cluster scores) among mechanically ventilated young and middle-aged EICU patients. After data cleaning, 500 valid cases were retained, with 400 used for model development and 100 held out as an independent test set.

A substantial symptom burden was observed at 1 month after discharge: the mean PCL-5 total score was 38.0 ± 9.1, and 70.8% of patients met the screening threshold for suspected PTSD (PCL-5 ≥ 33).

Consistent with symptom-cluster profiling, avoidance symptoms showed the highest mean item score among the four clusters, suggesting avoidance-related distress may be particularly prominent in this population.

### 5.2 Predictive performance and added value of the CNN-Attention framework

On the independent test set, the CNN-Attention model achieved an R^2^ of 0.804 for the PCL-5 total score (RMSE = 2.164; MAE = 1.935; MAPE = 7.43%), while the four symptom-cluster predictions showed R^2^ values ranging from 0.817 to 0.929.

More granularly, avoidance symptom prediction was the most accurate (R^2^ = 0.884), whereas the total score prediction was relatively weaker, a pattern that aligns with the model’s explicit cross-output constraint (total score = sum of subscale scores) that can propagate and accumulate errors into the total-score channel.

A key methodological contribution is the integration of dual attention mechanisms (channel and spatial attention) into a 1D-CNN designed for structured clinical features, coupled with a constraint term embedded into the loss function to enforce the arithmetic relationship among outputs.

The training process employed EarlyStopping and demonstrated smooth loss descent, with early termination around 542 epochs (loss≈0.0097), suggesting the constraint may stabilize optimization.

Importantly, the study benchmarked performance against multivariable linear regression, BP neural networks, a CNN model without attention, Random Forest, XGBoost, and the CNN-Attention model showed superior predictive accuracy on the test set, supporting the incremental value of attention for feature focusing in small-to-moderate, high-dimensional clinical datasets.

Compared with prior ICU-related PTSD prediction frameworks, which have largely relied on traditional regression models and binary PTSD outcomes [28], the present model was developed to predict continuous symptom severity and symptom-cluster profiles simultaneously. This distinction may be clinically relevant because binary classification alone may be insufficient for risk stratification, intensity-matched follow-up, and targeted intervention [28]. In addition, ICU-related PTSD is influenced by multiple interacting domains, including treatment invasiveness, acute illness trajectory, neuropsychiatric complications, and concurrent emotional distress [8,20,23–27], and such relationships may not be fully captured by conventional linear assumptions. By combining CNN-based feature extraction, attention-based feature weighting, and SHAP-based explanation, the present framework extends previous ICU-PTSD prediction approaches toward a more flexible and clinically informative modeling strategy.

### 5.3 Clinical interpretation of key predictors: mechanisms and plausibility

Explainability analyses using SHAP on the independent test set identified mechanical ventilation mode, admission urgency, ventilation duration, prior mental illness history, and delirium burden (CAM-ICU+ days) as the most influential contributors to the predicted PCL-5 total score. These findings are clinically coherent, as they jointly reflect (i) treatment invasiveness and perceived loss of control, (ii) acute severity and emergency context, and (iii) neuropsychiatric complications during critical illness.

Mechanical ventilation exposure (mode and duration). SHAP summary plots indicated that more invasive ventilation modes and longer ventilation durations tended to push predictions toward higher PTSD symptom severity. This is consistent with the clinical understanding that more intrusive respiratory support can intensify dyspnea-related fear, communication restriction, and helplessness—experiences that may consolidate traumatic memory traces.

Delirium burden. Delirium-positive days were positively associated with PTSD severity both in univariate comparisons and in SHAP-based model explanation, highlighting delirium as a potentially central risk pathway. The manuscript links delirium to fragmented, negative, and even delusional ICU memories, which plausibly amplify intrusive recollections and subsequent hyperarousal and avoidance.

Admission urgency and illness trajectory. Admission urgency and ICU length of stay were associated with higher PTSD scores, indicating that abrupt onset, intensive procedures, prolonged exposure to a high-stress environment, and sustained physiologic threat may cumulatively increase psychological trauma.

Psychiatric vulnerability and concurrent emotional distress. Prior mental illness history and the presence of anxiety/depression symptoms were influential predictors. The paper also emphasizes that anxiety is among the strongest and potentially modifiable risk factors, and that PTSD frequently co-occurs with anxiety and depression, warranting holistic assessment rather than isolated symptom screening. This supports the clinical utility of including HADS-A and HADS-D positivity as predictors for more actionable stratification.

Family support as a protective factor. SHAP results suggested that higher-quality/more frequent family visitation contributed negatively to predicted PTSD severity, consistent with the protective role of social support during and after critical illness. This implication is especially relevant for EICU contexts where separation, sensory deprivation, and uncertainty are common stress amplifiers.

### 5.4 Why multi-output symptom prediction matters

Prior ICU-PTSD prediction efforts frequently framed outcomes as binary PTSD presence/absence, which can be insufficient for clinical prioritization and intensity-matched follow-up planning. By predicting both the total PCL-5 score and symptom clusters, the present model supports nuanced profiling (e.g., prominent avoidance), which may better align with targeted intervention design. The observed prominence of avoidance symptoms in this cohort further underscores the potential benefit of dimension-aware prediction rather than sole reliance on total score thresholds.

In the test set, avoidance symptoms were predicted with the highest accuracy, which may reflect their relatively concentrated score range and/or stronger signal-to-noise ratio in the available predictors, though this requires confirmation in held-out independent test set.

### 5.5 Methodological considerations: Feature selection and interpretability

The study intentionally did not proceed to multivariable regression-based feature selection, arguing that (i) univariate screening sufficiently reduced dimensionality to 11 variables spanning multiple domains, (ii) CNN-Attention can model nonlinear interactions and handle multicollinearity without the constraints of traditional regression assumptions, and (iii) linear multivariable modeling may be poorly suited to complex nonlinear relationships between ICU exposures and PTSD severity.

This choice should be understood as a methodological trade-off rather than a procedural omission. Specifically, we prioritized predictive completeness over coefficient-level parsimony. A multivariable regression-based selection step might have produced a more compact set of predictors and improved the interpretability of individual coefficients under a linear framework; however, it also risked excluding variables that may contribute predictive value through nonlinear effects, interactions, or joint patterns that are difficult to detect using conventional regression assumptions. Given that the primary objective of this study was prediction rather than causal effect estimation, retaining clinically relevant candidate variables after preliminary univariate screening was considered a defensible strategy.

At the same time, the manuscript recognizes that deep learning models require dedicated interpretability tools; thus, SHAP was applied to enhance transparency and facilitate clinically meaningful interpretation.

### 5.6 Clinical implications and potential workflow integration

Given limited psychological care resources and the difficulty of diagnosing PTSD based on a single scale alone, a pragmatic risk prediction tool based on routinely obtainable clinical and psychological data may support earlier triage, stepped-care follow-up, and timely referral for comprehensive assessment.

Practically, the model could be deployed at discharge (or shortly after) to identify patients with high predicted total scores or specific high-risk symptom profiles (e.g., marked avoidance/hyperarousal). It may also help prioritize patients with high delirium burden, prolonged ventilation, invasive ventilation mode exposure, or concurrent anxiety/depression symptoms for proactive psychoeducation and structured follow-up.

### 5.7 Limitations

Several limitations should be considered when interpreting the findings of this study. First, although the study used a prospective observational cohort design, all participants were recruited from a single EICU in one tertiary hospital during a relatively short enrollment period. Therefore, the study population may reflect center-specific clinical practices, staffing patterns, sedation and visitation policies, and follow-up procedures. These factors may limit the generalizability of the findings to other hospitals, regions, and ICU settings. In addition, the study was restricted to young and middle-aged adults aged 18–59 years who survived to weaning, regained sufficient mental clarity, and were able to complete the required assessments. Older adults, patients with severe cognitive impairment, patients with acute psychiatric instability, and those who died, withdrew treatment, transferred to other institutions, or could not complete follow-up were not represented. As a result, the model may not be directly transportable to broader mechanically ventilated ICU populations or to patients with more severe neurological or psychiatric vulnerability.

Second, several measurement-related constraints should be acknowledged. PTSD symptom severity was assessed using the PCL-5 at 1 month after discharge. Although the PCL-5 is a validated and widely used instrument for symptom screening and severity assessment, it does not replace a structured diagnostic interview conducted by trained mental health professionals. Therefore, the outcome of this study should be interpreted as posttraumatic stress symptom burden rather than a definitive diagnosis of PTSD. The 1-month follow-up time point was selected to balance patient safety, data completeness, and the symptom window of the PCL-5; however, PTSD symptoms may fluctuate, persist, remit, or emerge later over the course of recovery. This study was therefore unable to characterize long-term symptom trajectories or delayed-onset PTSD. In addition, part of the psychological assessment and follow-up data was collected through self-report via WeChat or email, which may have introduced recall bias, response bias, or mode-of-administration differences.

Third, the modeling strategy also has methodological limitations. Although an independent internal test set was used to evaluate generalization, the model has not yet undergone external validation in independent multicenter cohorts. The sample size was acceptable for initial model development but remains modest for a deep learning framework, and the model may be sensitive to sampling variation, local case-mix characteristics, and predictor distributions. Candidate predictors were selected based on univariate screening, clinical relevance, and data completeness, which was consistent with the prediction-oriented objective of the study; however, this approach may have excluded variables with weak univariate associations but potentially meaningful nonlinear or interaction effects. Moreover, the 1D-CNN architecture required the 11 structured clinical variables to be arranged in a fixed order, and the assumption that neighboring variables contain informative local patterns may not fully reflect the structure of tabular clinical data. Although SHAP analysis was used to improve interpretability, SHAP values describe model-based feature contributions and should not be interpreted as causal effects.

Finally, the practical implementation of the proposed model remains to be evaluated. The current findings suggest that the model may support early risk stratification and follow-up planning, but its clinical utility depends on external validation, calibration across settings, integration with electronic medical record systems, clinician acceptance, and the availability of psychological assessment and referral pathways. Importantly, the model should be regarded as a decision-support tool for identifying patients who may benefit from further psychological evaluation, rather than as a standalone diagnostic instrument. Future multicenter prospective studies should validate and recalibrate the model, examine its performance in older and more heterogeneous ICU populations, incorporate additional modifiable clinical exposures, and determine whether model-informed stepped-care follow-up improves long-term psychological outcomes.

### 5.8 Future directions

Future studies should prioritize: (i) multi-center prospective validation and recalibration; (ii) extension to older populations and diverse ICU settings; (iii) incorporation of additional modifiable exposures (e.g., sedation/analgesia dosing, ICU diary use, sleep disruption metrics) to improve both performance and intervention linkage; and (iv) pragmatic trials to evaluate whether model-informed stepped-care follow-up reduces long-term psychological morbidity and improves quality-of-life outcomes.

## 6 Conclusion

This study developed a CNN-Attention multi-output regression model using routinely obtainable clinical and psychological variables to predict PCL-5 total and symptom-cluster scores at 1 month after discharge in mechanically ventilated young and middle-aged EICU patients.

The model demonstrated strong generalization on an independent test set (PCL-5 total score R^2^ = 0.804; symptom-cluster R^2^ up to 0.929) and outperformed traditional regression, BP neural networks, and a CNN model without attention.

Explainability analyses highlighted ventilation exposure (mode and duration), admission urgency, delirium burden, psychiatric vulnerability, and concurrent anxiety/depression as key contributors, while family visitation appeared protective.

Collectively, these findings suggest that the proposed model may support early risk stratification, targeted follow-up, and individualized psychological care planning in resource-constrained EICU settings, although larger multi-center prospective validation and workflow impact studies remain necessary.

## Supporting information

S1 Table EICU mechanically ventilated patients: baseline characteristics (n = 400). (DOCX)

S1 Text Terms and abbreviations. (DOCX)

S1 Checklist STROBE checklist. This checklist is based on the STROBE Statement, which is licensed under the Creative Commons Attribution 4.0 International License (CC BY 4.0). Source: STROBE Statement, https://www.strobe-statement.org/.
(DOCX)

S1 Data De-identified minimal dataset underlying the findings of this study. (RAR)
