# Supplementary material for: Discharge-time prediction of 1-month posttraumatic stress symptom severity (PCL-5) after mechanical ventilation using a dual-attention 1D-CNN: Development and validation
Source: PLOS Ment Health. 2026 Jun 9;3(6):e0000629. doi: 10.1371/journal.pmen.0000629 (PMC13249175; doi:10.1371/journal.pmen.0000629)
Supplement: S1 Table — (DOCX) [file pmen.0000629.s001.docx]

S1 Table. EICU Mechanically Ventilated Patients: Baseline Characteristics（n=400）

| Variable | Category | Frequency (n) | Percentage（%） |
| --- | --- | --- | --- |
| Gender | Male | 203 | 50.8 |
|  | Female | 197 | 49.2 |
| Age | 18~28 | 42 | 10.1 |
|  | 29~50 | 248 | 62.5 |
|  | 51~59 | 110 | 27.4 |
| Marriage | Married | 284 | 71.0 |
|  | Unmarried | 83 | 20.8 |
|  | Divorced or widowed | 33 | 8.2 |
| Career | Stable employment | 160 | 40.0 |
|  | Unstable employment | 150 | 37.5 |
|  | Flexible employment | 90 | 22.5 |
| Educational level | Junior high school or below | 155 | 38.8 |
|  | Senior high school or secondary technical school | 163 | 40.7 |
|  | College level or above | 82 | 20.5 |
| Frequency and quality of family visits | High frequency with good quality | 178 | 44.5 |
|  | Low frequency or poor quality | 152 | 38.0 |
|  | No visits | 70 | 17.5 |
| Disease related family burden | Very severe | 110 | 27.5 |
|  | Moderate | 190 | 47.5 |
|  | Mild | 100 | 25.0 |
| Medical expenses | ＜100000 | 145 | 36.2 |
|  | 100000～150000 | 140 | 35.0 |
|  | ＞150000 | 115 | 28.8 |
| Chronic underlying disease | Yes | 153 | 38.2 |
|  | No | 247 | 61.8 |
| History of mental illness | Yes | 33 | 8.3 |
|  | No | 367 | 91.7 |
| Urgency of admission | Level 1 (extreme urgency) | 123 | 30.8 |
|  | Level 2 (urgent) | 154 | 38.5 |
|  | Level 3 (moderate urgency) | 85 | 21.2 |
|  | Level 4 (non-urgent) | 38 | 9.5 |
| APACHE II score | 0～9 | 49 | 12.2 |
|  | 10～19 | 191 | 47.8 |
|  | 20～29 | 127 | 31.8 |
|  | ＞30 | 33 | 8.2 |
| Surgery during hospitalization | Yes | 169 | 42.3 |
|  | No | 231 | 57.7 |
| Length of stay in EICU | ≤5 | 132 | 33.0 |
|  | 6~10 | 140 | 35.0 |
|  | ＞10 | 128 | 32.0 |
| Total length of hospital stay | ≤10 | 154 | 38.5 |
|  | 11~20 | 136 | 34.0 |
|  | ＞20 | 110 | 27.5 |
| Cumulative days of delirium (CAM-ICU ) | 0 | 170 | 42.5 |
|  | 1~2 | 150 | 37.5 |
|  | ≥3 | 80 | 20.0 |
| Primary reason for mechanical ventilation | Respiratory failure (central / peripheral) | 112 | 28.0 |
|  | Surgery related (post general anesthesia / cardiothoracic surgery, etc.) | 120 | 30.0 |
|  | Trauma related (chest trauma / traumatic brain injury, etc.) | 92 | 23.0 |
|  | Other (post cardiopulmonary resuscitation / poisoning, etc.) | 76 | 19.0 |
| Mode of mechanical ventilation | Non-invasive | 120 | 30.0 |
|  | Invasive | 210 | 52.5 |
|  | Both | 70 | 17.5 |
| Duration of mechanical ventilation | ≤72h | 226 | 56.5 |
|  | ＞72h | 174 | 43.5 |
| Level of consciousness before mechanical ventilation | Awake (GCS 13 – 15) | 222 | 55.5 |
|  | Drowsy (GCS 9 – 12) | 102 | 25.5 |
|  | Coma (GCS 3 – 8) | 76 | 19.0 |
| Memory during mechanical ventilation | Full recall | 63 | 15.7 |
|  | Partial recall | 216 | 54.0 |
|  | No recall | 121 | 30.3 |
| Use of sedatives | Yes | 268 | 67.0 |
|  | No | 132 | 33.0 |
| Received CRRT / ECMO treatment | Yes | 112 | 28.0 |
|  | No | 288 | 72.0 |
| Anxiety positive (HADS-A ≥ 8) | Yes | 295 | 73.7 |
|  | No | 105 | 26.3 |
| Depression positive (HADS-D ≥ 8) | Yes | 267 | 66.8 |
|  | No | 133 | 33.3 |
