# Supplementary material for: Discharge-time prediction of 1-month posttraumatic stress symptom severity (PCL-5) after mechanical ventilation using a dual-attention 1D-CNN: Development and validation
Source: PLOS Ment Health. 2026 Jun 9;3(6):e0000629. doi: 10.1371/journal.pmen.0000629 (PMC13249175; doi:10.1371/journal.pmen.0000629)
Supplement: S1 Text — (DOCX) [file pmen.0000629.s002.docx]

S1 Text: Terms and Abbreviations

| Item | Abbreviation |
| --- | --- |
| Mechanical Ventilation | MV |
| Frequency and Qualityof Family Visitation | Fam Visit Freq & Qual |
| History of Mentallllness | Mental lll Hist |
| Admission Urgency | Adm Urgency |
| Length of Stay in ElCU (days) | EICU LOS |
| Cumulative Days of Delirium (CAM-ICU+) | Del(CAM-ICU+) Days |
| Mode of Mechanical Ventilation | MV Mode |
| Duration of Mechanical Ventilation | MV Duration |
| Receipt of CRRT/ECMO Treatment | CRRT/ECMO Tx |
| Anxiety Positive (HADS-A ≥8) | Anxiety (HADS-A≥8) |
| Depression Positive(HADS-D ≥8) | Depression(HADS-D≥8) |
| Intrusive Symptoms | IS |
| Avoidance Symptoms | AS |
| Negative Alterations in Cognition and Mood | NACM |
| Alterations in Arousa and Reactivity | AAR |
