# Supplementary figures and images for: Discharge-time prediction of 1-month posttraumatic stress symptom severity (PCL-5) after mechanical ventilation using a dual-attention 1D-CNN: Development and validation
Source: PLOS Ment Health. 2026 Jun 9;3(6):e0000629. doi: 10.1371/journal.pmen.0000629 (PMC13249175; doi:10.1371/journal.pmen.0000629)

**S1 Checklist. STROBE guideline adherence statement**


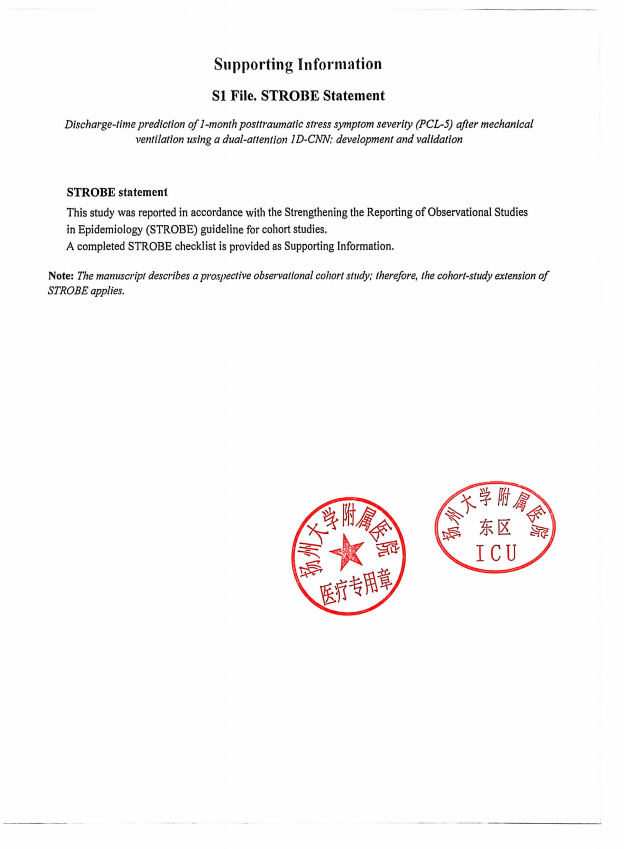


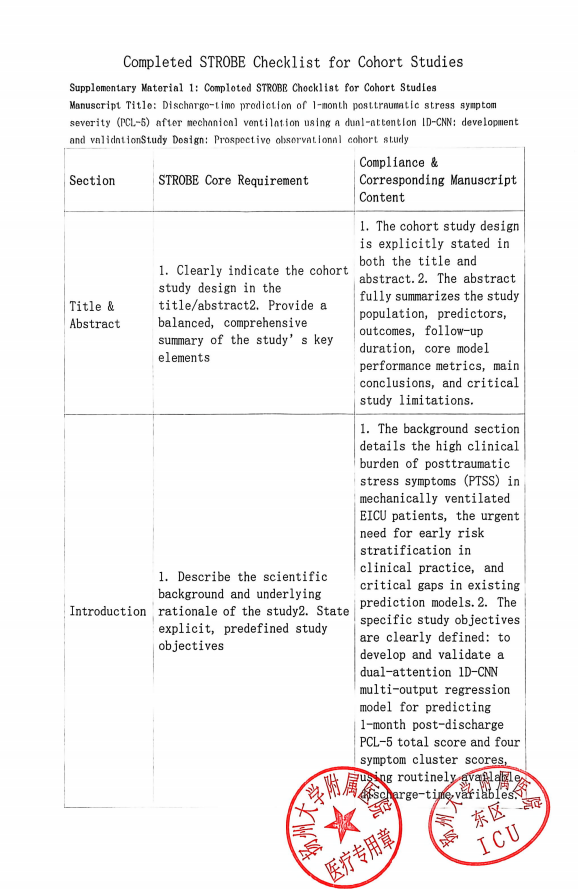


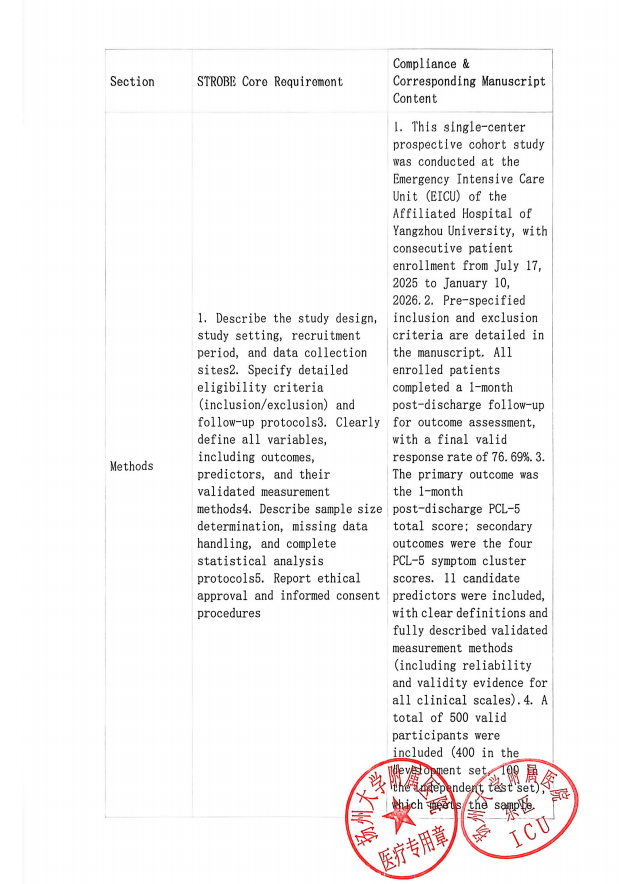


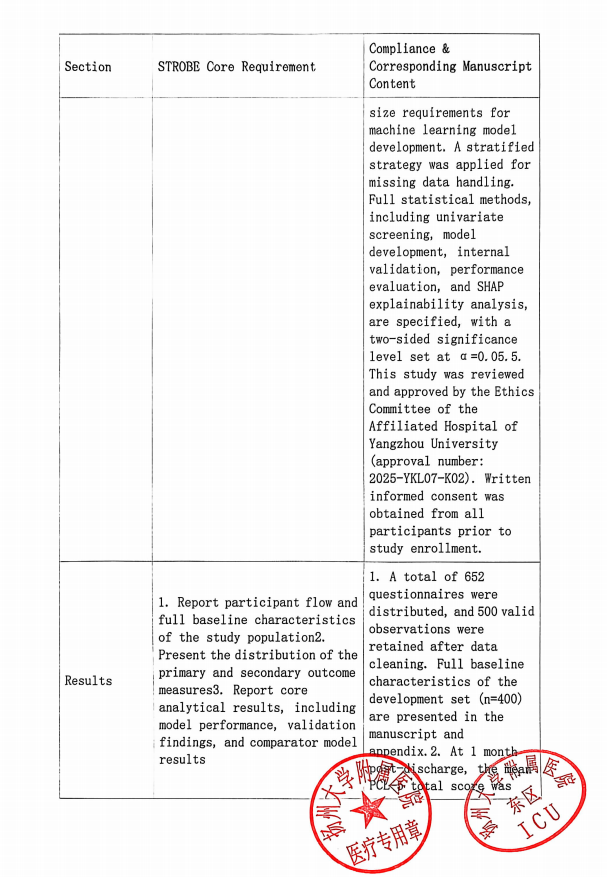


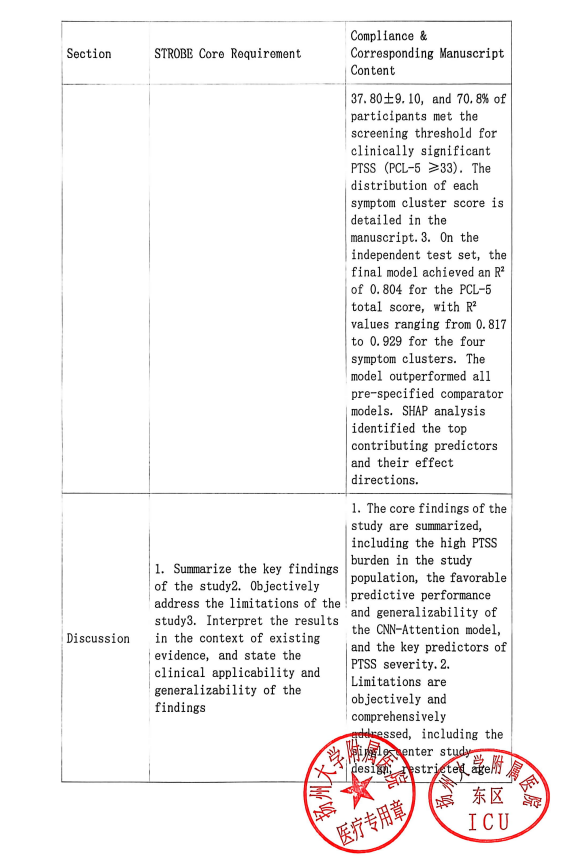


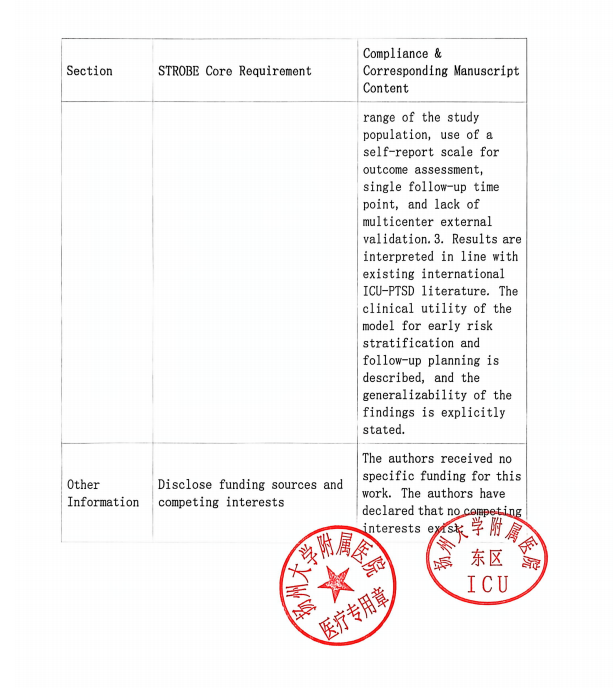

Supplement: S1 Checklist — This checklist is based on the STROBE Statement, which is licensed under the Creative Commons Attribution 4.0 International License (CC BY 4.0). Source: STROBE Statement, https://www.strobe-statement.org/. (DOCX) [file pmen.0000629.s003.docx]
